# Time to Detection of Growth for *Mycobacterium tuberculosis* in a Low Incidence Area

**DOI:** 10.3389/fcimb.2021.704169

**Published:** 2021-08-19

**Authors:** Rechana Vongthilath-Moeung, Antoine Poncet, Gesuele Renzi, Jacques Schrenzel, Jean-Paul Janssens

**Affiliations:** ^1^Division of Pulmonary Diseases, Geneva University Hospitals, Geneva, Switzerland; ^2^Center for Clinical Research & Division of Clinical-Epidemiology, Department of Health and Community Medicine, University of Geneva, University Hospitals of Geneva, Geneva, Switzerland; ^3^Bacteriology Laboratory, Division of Laboratory Medicine, Geneva University Hospitals, Geneva, Switzerland; ^4^Division of Infectious Diseases, Geneva University Hospitals, Geneva, Switzerland

**Keywords:** mycobacteria, *Mycobacterium tuberculosis*, time to detection of growth, mycobacterium growth indicator tube, nuclear acid amplification techniques

## Abstract

**Background:**

Diagnosis of *Mycobacterium tuberculosis* (MTB) infection can be confirmed by Xpert assays within hours. However, when sample size does not allow performing both culture and Xpert, or if Xpert is negative, then formal diagnosis of MTB relies on culture and time to detection of growth (TDG) becomes critical for clinical management.

**Objectives:**

To determine TDG in Xpert negative samples, or in samples in which Xpert could not be performed, in a low-incidence area for MTB.

**Methods:**

Retrospective analysis (2015-2020) of a database including all cultures for mycobacteria in a University Hospital covering approximately 500’000 inhabitants. Analysis was restricted to culture positive (C+) samples for MTB for which 1/Xpert was negative or could not be performed because of limited sample volume, and 2/collected from subjects treated less than 24 hours. TDG was analyzed according to microscopy, origin of sample (pulmonary or not) and presence of cavitation.

**Results:**

Among 837 C+ samples for MTB, 236 samples (80% of respiratory origin) from 147 patients fulfilled study criteria; 78 samples (49 patients, 33%) were acid-fast bacilli (AFB) positive. Median (IQR) TDG was 25 (17; 40) days for all samples. TDG exceeded 28 days in 43% of samples and was significantly shorter in AFB+ *vs* AFB- samples, and samples from cavitary *vs* non cavitary or extra-thoracic disease.

**Conclusions:**

In Xpert negative samples, or samples for which Xpert could not be performed, TDG exceeded 4 weeks in 43% of samples. AFB+ and samples from cavitary lung disease had a significantly shorter TDG.

## Introduction

Since the publication by Boehme et al. of the contribution of rapid molecular diagnostic techniques for detecting TB (referred to as NAAT: Nuclear acid amplification techniques), and their rapid implementation worldwide, the diagnosis of tuberculosis has changed dramatically (Boehme et al., 2010). NAAT have not only increased the analytical sensitivity as compared to microscopy (auramine stain and Ziehl-Neelsen for acid-fast bacilli: AFB): they allow a positive diagnosis of tuberculosis within hours, while requiring only basic levels of laboratory skills. Multicentric studies in high prevalence areas show that NAAT allow early identification in approximately 80% of all culture positive TB cases irrespective of microscopy (Theron et al., 2014; Calligaro et al., 2017). Boehme et al. showed in a large multicentric field study that Xpert/MTB-RIF could detect 90.3% of culture positive samples (76.9% in smear negative samples) (Boehme et al., 2011). Because of the low skills and the minimal hands-on time involved, there is a trend towards favoring the use of NAAT instead of microscopy: indeed, in some centers, microscopy for detecting AFB may not always be available, or not on the spot. The present contribution of microscopy is mainly to assess risk of transmission, a role that may in a near future, be replaced by sensitive quantitative PCR assays.

In patients with clinical suspicion of MTB, a certain number of cases remain NAAT negative (or samples are insufficient to perform NAAT and culture): in these cases, diagnosis relies on a positive identification by culture, since, in low incidence settings, microscopy alone cannot formally discriminate between MTB and nontuberculous mycobacteria (NTM). Time to detection of growth (TDG) in liquid and/or solid media is then critical to determine when a case of TB can either be confirmed or reasonably excluded.

While time to culture conversion (i.e.: time to negative culture after initiating tuberculostatic treatment) is frequently reported and used as a surrogate marker for response to treatment (Olaru et al., 2014), recent data concerning TDG in samples taken before initiation of treatment are scarce. TDG is inversely correlated with number of acid-fast bacilli (AFB) on sputum smears (Perrin et al., 2007; Bark et al., 2011; Olaru et al., 2014), number of colony forming units in sputum specimens (Diacon et al., 2014) and risk of relapse after therapy in clinical trials (Bark et al., 2012). Baseline TDG is also correlated to time to conversion (Visser et al., 2012) and inversely related to transmissibility (Khan et al., 2018). TDG in treatment-naïve subjects are, as expected, lower in smear positive and NAAT positive subjects (Khan et al., 2018) with median values ranging between 5 (Bisognin et al., 2020; Ma et al., 2020) and 23 days (Boehme et al., 2011; Bark et al., 2012; Pfyffer and Wittwer, 2012; Tyrrell et al., 2012; Visser et al., 2012; Olaru et al., 2014; Ogwang et al., 2015; Diriba et al., 2017; Khan et al., 2018; Zhao et al., 2019). A few recent studies suggest that, in the majority of clinical samples, growth of MTB could be detected within 28 days of incubation (Pfyffer and Wittwer, 2012; Ogwang et al., 2015). A US multicentric study reported a median TDG of 14 days (using the Mycobacterium Growth Indicator Tube (MGIT) system, Becton Dickinson, Allschwil, Switzerland) with a 100% detection of all positive samples within 28 days, thus suggesting that there was no additional benefit of growing MTB on solid or liquid media for more than 5 weeks (Tyrrell et al., 2012).

The aims of this study were: 1/to quantify, in a real-life low TB incidence clinical setting, time to detection of growth for MTB and 2/to determine if 4 weeks were sufficient to detect all or most MTB growth in patients for whom a diagnosis could not be established by Xpert/MTB-RIF assays. Because of the systematic implementation of Xpert techniques in our area, we restricted our analysis to culture positive clinical samples identified as MTB for which 1/Xpert/MTB-RIF was negative or could not be performed because of limited sample volume, and 2/collected from subjects either naïve to tuberculostatic drugs or treated for less than 24 hours. Duration of TDG was analyzed according to results of microscopy when performed, site of infection and presence of cavitation in pulmonary TB.

## Patients and Methods

This study was performed at Geneva University Hospitals (HUG), a 2008-bed complex providing care for a population of approximately 500’000 inhabitants. Switzerland has a low incidence for TB (7/10^5^ inhabitants). All clinical samples sent for microbiological analysis within HUG – whether provided by inpatients or outpatients - are treated only by the Bacteriology Laboratory of HUG. An online database is kept up-to-date and includes all samples processed by the laboratory.

We analyzed all clinical samples with positive cultures for mycobacteria included in this database over a 5-year period (2015-2020). This report focuses only on mycobacteria belonging to the *M. tuberculosis complex* (MTB).

The following items were recorded from the electronic patient files: age, gender, site of infection (when pulmonary, we recorded presence of cavitation), presence and type of immunosuppression, relevant comorbidities, results of microscopy, and MTB sub-species identified. Details of treatment and treatment outcome are not reported.

The study protocol was approved by our local ethics committee (CCER 2020-00872) and registered at Clinicaltrials.gov (NCT04718571).

### Processing of Clinical Samples

Samples sent for detection of mycobacteria are processed as follows, according to WHO/CDC guidelines:

Culture is favored over direct testing (PCR, microscopy) whenever the sample volume is limited. When the sample volume is sufficient, direct testing is performed using the Xpert MTB/RIF ULTRA on the GeneXpert System (Cepheid) for the detection of *Mycobacterium tuberculosis* and of potential mutations in the *rpoB* gene conferring rifampin-resistance. Likewise, a direct microscopic examination is performed using first an auramine based stain followed by a Ziehl-Neelsen stain. Cultivation of samples is performed on a combination of liquid and solid media: samples are inoculated into Mycobacterium Growth Indicator Tube (MGIT) bottles (MGIT 960, Becton Dickinson, Allschwil, Switzerland) and on the following two solid media: Coletsos (Bio-Rad, Marnes-la-Coquette, France) and Loewenstein-Jensen (Becton Dickinson), all of which are incubated for 12 weeks. Whenever positive, the supernatant of the MGIT bottle is subjected to an immunochromatographic assay detecting protein MPT64 (Becton Dickinson), as a surrogate marker for the identification of *Mycobacterium tuberculosis*. Supernatant is also inoculated on two separate solid media: Coletsos (bioRad) and Loewenstein-Jensen (Becton Dickinson), as described above, for further characterization.

Solid cultures permit further identification by using the Xpert MTB/RIF ULTRA for detecting *M. tuberculosis* or by *rpoB* gene sequencing for the identification of NTM.

### Inclusion/Exclusion of Samples

Samples sent for culture of mycobacteria came from inpatient and outpatient hospital sites, all within HUG. Bronchial aspirates and bronchoalveolar lavages are also routinely cultivated for mycobacteria in our institution, irrespective of whether there is or not a clinical suspicion of tuberculosis or NTM infection.

Were excluded from the analysis: 1/external quality control strains; 2/all positive cultures for NTM; 3/all samples for which Xpert MTB/RIF ULTRA was positive for *M. tuberculosis complex*; 4/all samples collected more than 24 hours after introduction of anti-TB treatment.

Thus, we included only: samples with 1/a positive culture for *M. tuberculosis complex, and* 2/either a negative or no Xpert/MTB-RIF, irrespectively of direct microscopy.

### Statistical Analysis

Descriptive data are reported for patient characteristics, type and origin of samples, and results of microscopy according to clinical presentation. Quantitative variables were described as median and interval interquartile (IQR) and qualitative variables were described as counts, percentages.

Time to detection of growth (TDG) is reported as the time between inoculation to liquid and solid media, and first signal of growth. TDG was described overall, according to results of microscopy (presence or absence of AFB) and according to case presentation (cavitary *vs.* non cavitary pulmonary tuberculosis, *vs.* extra-pulmonary tuberculosis). Descriptive statistics of TDG included mean (SD), median (IIQ), 90^th^ centile, and counts and percentages of samples for which TDG was > 28 days. Distribution of TDG was represented using Empirical cumulative distribution function (ECDF) plots and compared between groups (positive *vs.* negative microscopy findings; cavitary *vs.* non cavitary pulmonary tuberculosis *vs.* extra-thoracic tuberculosis) using linear regression models (samples were considered as independent observations).

Statistical significance was assessed at the two-sided 0.05 level for all analyses.

All analyses were performed using R software version R-4.0.2 (R Foundation for Statistical Computing, Vienna, Austria. URL https://www.R-project.org/).

## Results

**Figure 1:**
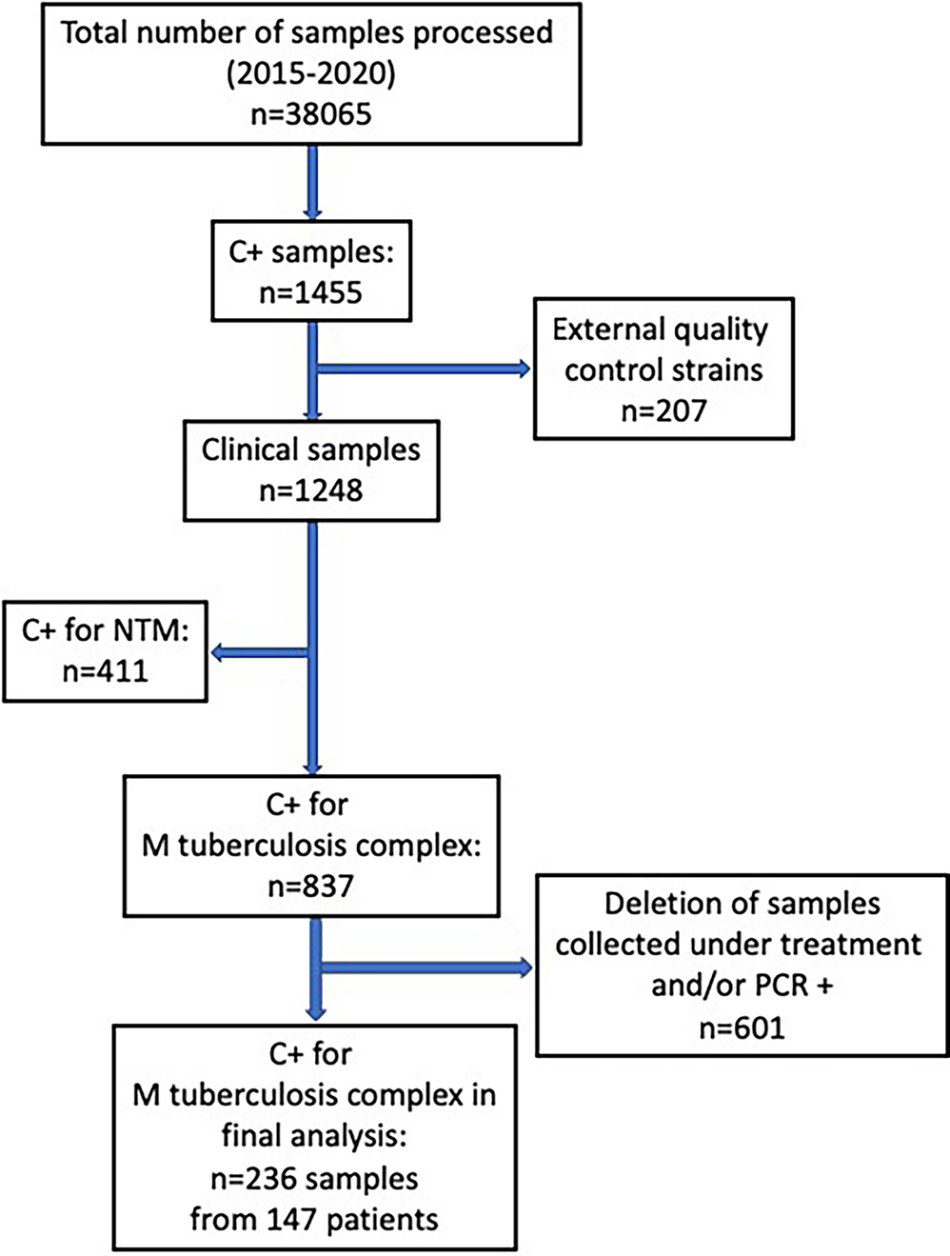
Flow-chart of samples analyzed from the database of all positive culture results for mycobacteria at our institution over a 5-year period (May 2015- May 2020).

A total of 236 samples were analyzed, originating from 147 patients (**Figure 1**). Number of samples per patient ranged from 1 to 5 (Mean: 1.6; SD: 0.9). All strains were identified as *M. tuberculosis* except for one *M. africanum*, and four *M. bovis*.

Xpert was performed on 96 samples (41%). Of all samples in which Xpert was performed (and negative), 6/96 showed AFB on microscopic examination (all confirmed as *M. tuberculosis* by culture, all of pulmonary origin) yielding 6.3% of false negative Xpert results.

**Table 1** shows baseline characteristics of the study population, main site of infection, comorbidities, factors of immunosuppression, origin, and resistance profile to first line tuberculostatic drugs. Samples came from a relatively young population, mostly of foreign origin (only 24% were from Western Europe including Switzerland) with few comorbidities, or factors of immunosuppression. Isolated resistance to tuberculostatic drugs or MDR strains were rare (n=14, 9%).

**Table 1:** Patients’ characteristics (n=147 patients).

	All patients (n=147)
Female, *n (%)*	58 (39)
Age (years)	
Median (IQR)	36 (25; 46)
Range	3 to 94
*Comorbidities, n (%)*	25 (17)
Diabetes	10 (7)
Cardiac disease	4 (3)
Chronic obstructive pulmonary disease	3 (2)
Bronchiectasis	1 (1)
Chronic renal disorder	1 (1)
Hepatic disorder	1 (1)
Alcohol abuse	2 (1)
Other	3 (2)
*Immunosuppression, n (%)*	14 (10)
Human immunodeficiency virus co-infection	4 (3)
Systemic glucocorticosteroids	2 (1)
Other	7 (5)
*Origin, n (%)*	
Africa	67 (46)
Western Europe*	33 (22)
Eastern Europe	15 (10)
Asia	19 (13)
South America	10 (7)
Missing	3 (2%)
*Resistance to first line tuberculostatic drugs, n (%)*	
Any resistance	14 (9)
Isoniazide alone	6 (4)
Rifampicine alone	2 (1)
Ethambutol alone	0
Pyrazinamide alone	2 (1)
Multidrug resistance	4 (3)

*includes Switzerland.

**Table 2** shows the source of the 236 *samples*: 80% of all samples (n=189) were of respiratory origin, and 20% (n=47) were extra-pulmonary. Microscopy was performed on 230 samples (97.5%) and was positive for AFB on 78 samples (34%), and negative on 152 samples (66%); in 6 cases microscopy was not performed due to insufficient sample volume (**Table 3**).

**Table 2:** Origin of samples (n=236 samples).

	All strains (n=236)	Cavitary pulmonary TB	Non-cavitary Pulmonary TB
**Pulmonary samples, n (%)**	**189 (80)**	**73 (31)**	**116 (49)**
Sputum, n	136	65	71
Bronchial aspirate, n	25	5	20
BAL, n	11	3	8
Intra-thoracic lymph nodes aspirate *n*	9	0	9
TBB, lung biopsy	8	0	8
**Extra pulmonary samples, n (%)**	**47 (20)**		
Soft tissue, n*	11		
Extra-thoracic lymph nodes aspirate*, n*	10		
Pleura, n**	11		
Urine aspirate*, n*	6		
Gastric aspirate*, n*	5		
CSF*, n*	2		
Joint fluid*, n*	1		
Unspecified	1		

BAL, Broncho-alveolar lavage; TBB, trans-bronchial biopsies; CSF, cerebro-spinal fluid; *non-pulmonary tissues samples from skin or surgical sites; **Pleura: includes 1 pleural biopsy.

**Table 3:** Results of microscopy, overall and by clinical presentation.

	All	Positive microscopy	Negative microscopy	Microscopy not performed
Number of patients, n (%)	n=147	n=49	n=96	n=2
Non-cavitary pulmonary TB	63 (43)	11 (22)	51 (52)	1 (50)
Cavitary pulmonary TB	48 (33)	38 (78)	10 (10)	–
Extra-pulmonary TB	36 (24)	–	35 (36)	1 (50)
Number of samples, n (%)	n=236	n=78	n=152	n=6
Respiratory samples from non-cavitary TB	116 (49)	15 (19)	100 (66)	1 (17)
Respiratory samples from cavitary TB	73 (31)	56 (72)	16 (10)	1 (17)
Extra-pulmonary samples	47 (20)	7 (9)	36 (24)	4 (67)

TB, tuberculosis.

**Figure 2:**
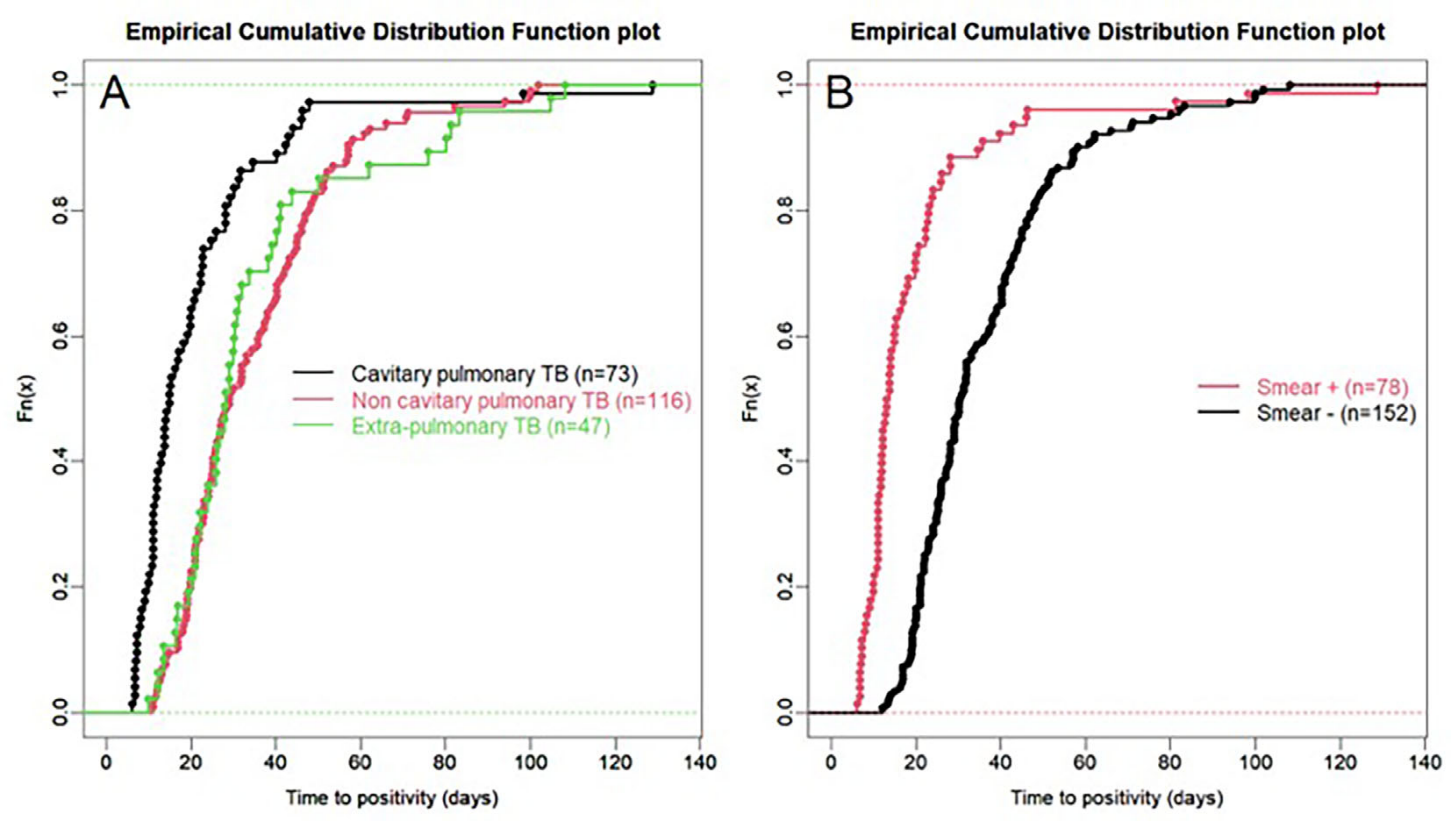
**(A)** Empirical cumulative distribution function plot of time to detection of growth in samples according to case presentation: cavitary *vs.* non-cavitary pulmonary tuberculosis *vs.* extra-pulmonary tuberculosis. **(B)** Empirical cumulative distribution function plot of time to detection of growth in samples according to microscopy (positive i.e. presence of acid-fast bacilli (AFB), *vs* negative: absence of AFB) irrespective of case presentation. All samples are PCR negative.

**Figure 3:**
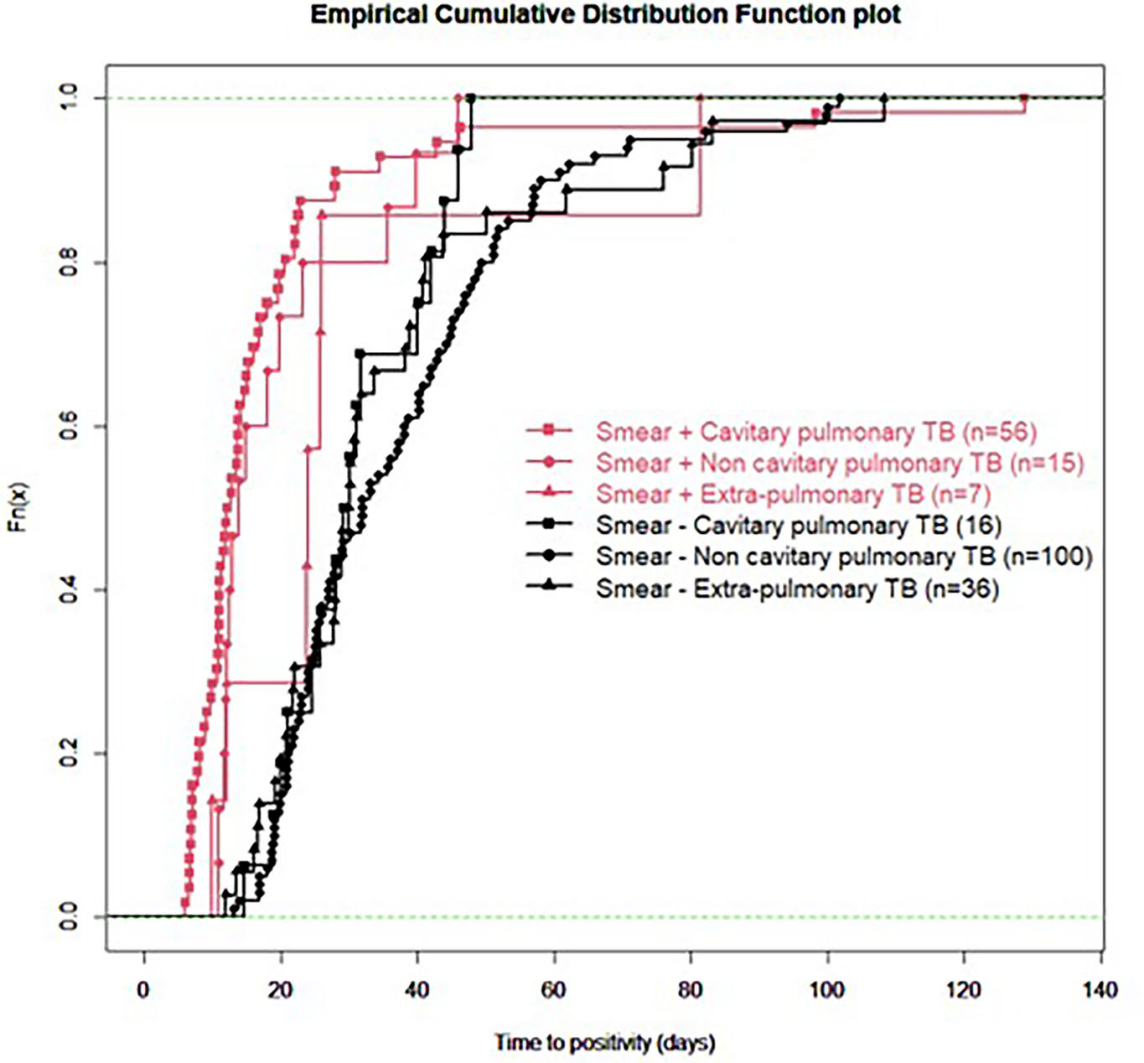
Empirical cumulative distribution function plot of time to detection of growth in samples according to microscopy (positive i.e. presence of acid-fast bacilli (AFB), *vs* negative: absence of AFB) and clinical presentation (cavitary *vs.* non-cavitary pulmonary tuberculosis *vs.* extra-pulmonary tuberculosis). All samples are PCR negative.

For all samples, median (IQR) time to detection of growth (TDG) of M tuberculosis complex was 25 days (IQR: 17; 40); range: 6-129 days; 90^th^ centile: 55 days. For 101 samples (43%), time to detection of growth exceeded 28 days. **Table 4** provides TDG according to result of clinical presentation, and microscopy. **Figure 2** shows TDG distribution according to A) case presentation (cavitary *vs.* non cavitary pulmonary tuberculosis, *vs.* extra-thoracic tuberculosis, p<0.001) and B) microscopy (positive i.e. presence of acid-fast bacilli (AFB), *vs* negative: absence of AFB, p>0.001). **Figure 3** shows TDG distribution according to both items combined.

**Table 4:** Time to detection of growth (TDG) according to results of microscopy and clinical presentation.

Time to detection of growth (in days)	Mean (SD)	Median (IQR)	90^th^ centile	TDG>28 days, n (%)	P value^1^
*All samples (n=236)*	31 (21)	25 (17 to 40)	55	101 (43)	
*Case description*					<0.001
Respiratory samples from non-cavitary TB (n=116)	35 (19)	29 (21 to 45)	57	61 (53)	
Respiratory samples from cavitary TB (n=73)	21 (19)	15 (11 to 25)	42	16 (22)	
Extra-pulmonary samples (n=47)	35 (24)	28 (21 to 40)	78	24 (51)	
*Smear*					<0.001
Positive (n=78)	20 (19)	13 (11 to 22)	35	10 (13)	
Negative (n=152)	36 (19)	30 (23 to 44)	58	90 (59)	

TB, tuberculosis; ^1^inear regression model.

## Discussion

In this retrospective study, we aimed to establish, in a real-life setting, what was the time to detection of growth (TDG) in liquid and/or solid growth media for *M. tuberculosis complex* for clinical samples which were either Xpert negative or for which Xpert could not be performed because of sample size and/or quality. We excluded from our analyses samples from patients who had received tuberculostatic treatment for more than 24 hours to minimize impact of drugs on TDG. Our results show that in this specific group of clinical samples, median TDG was 25 (IQR: 17; 40) days. In 43% of samples studied, TDG exceeded 28 days. Cavitation, pulmonary *vs* extra-pulmonary samples and presence of acid-fast bacilli (AFB) on microscopy had a significant impact on TDG (**Figures 2**, **3**, **Table 4**).

The rationale of this selection of samples was as follows: 1/large scale implementation of Xpert/MTB-RIF has increased same-day diagnosis, with a diagnostic sensitivity exceeding 80% in culture positive samples, irrespective of microscopy (Boehme et al., 2011; Khan et al., 2018); 2/in patients who do not have a same-day diagnosis by Xpert/MTB-RIF (negative test or not performed because of sample size), and who will eventually become culture positive, TDG determines when a diagnosis MTB can either be confirmed (as well as drug sensitivity profile), or reasonably excluded (treatment will most often have been started based on clinical suspicion). Our focus was therefore on TDG for all culture positive clinical samples fulfilling these criteria.

In this study, TDG exceeded the 4-5 week duration reported by several authors as sufficient for identifying all culture positive samples (Pfyffer and Wittwer, 2012; Tyrrell et al., 2012; Ogwang et al., 2015; Khan et al., 2018). Indeed, 90^th^ centile for TDG of all samples was 55 days, and even 58 days in smear negative cases. There are several possible explanations for this. Firstly, both presence of AFB on microscopy and positive Xpert assay results are suggestive of a higher organism burden, which is associated with a shorter TDG (Tyrrell et al., 2012; Visser et al., 2012; Diriba et al., 2017). In this study, all samples were all either Xpert/MTB-RIF negative, or could not be processed for Xpert/MTB-RIF; furthermore, 64% of all samples did not show AFB on microscopic examination. This suggests that the samples selected probably had a low organism burden, after excluding all cases for which a rapid diagnosis had been provided by Xpert/MTB-RIF. Secondly, 20% of all samples were extra-pulmonary: these samples have a lower number of bacilli, which can impact on TDG (Mazza-Stalder et al., 2012). This selection was deliberate, to conform with the diagnostic process since Xpert/MTB-RIF systems are systematically used. Our data do not support therefore the assumption that 4-5 weeks suffice to exclude the possibility of a positive culture: when empirical treatment is initiated, and if being culture negative is decisive to interrupt the treatment, then treatment should be pursued for at least 2 months before considering interrupting tuberculostatic drugs.

The fact that 33% of all samples (and patients) were smear positive for AFB could be considered as a confirmation of MTB. However, we did not delete these samples from our analyses for the following reasons. On-the spot immediate microscopy (auramine and Ziehl-Neelsen stains for AFB) is not available in all centers, and requires expertise, as opposed to Xpert assays. Also, in a low incidence country such as Switzerland (7/10^5^ inhabitants), with NTM cases being more and more frequently identified in clinical practice as in neighbor countries (Hoefsloot et al., 2013; Ringshausen et al., 2016; Vongthilath-Moeung et al., 2021), presence of AFB cannot be considered as *diagnostic* of MTB although it does increase the probability of MTB. Our analyses reflect a pragmatic approach in which microscopy may in a near future play a decreasing role in diagnosing MTB.

The present data show very clearly that TDG is significantly longer in AFB negative *vs* positive samples (Median, IQR: 30 (23; 44) *vs* 13 (11; 22) days), and 59% of AFB negative samples had a TDG > 28 days. Similar differences were found between samples from cavitary pulmonary TB *vs* samples from non-cavitary or extra-thoracic TB (**Table 4** and **Figures 2**, **3**). TDG for samples from non-cavitary or extra-thoracic TB were similar. This information is therefore crucial for clinicians to anticipate TDG.

This study has a few limitations. Firstly, it is based on a retrospective analysis of laboratory data, and although the data are comprehensive and include all samples processed during the 5-year period defined, recording errors cannot be excluded. Secondly, we do not know how many samples were sent per patient all together, and how many negative results occurred for each patient, and thus we can only comment on TDG in culture positive samples with little or no impact of tuberculostatic treatment. This is a *sample-based* and not a *patient-based* analysis. Data provided suggest that, if Xpert/MTB-RIF is negative or unavailable, 43% of samples collected within the first 24 hours of an empirical treatment will become positive after more than 4 weeks. It will be important through future studies to determine at a national and local level what is the present percentage of cases which are diagnosed only by culture or treated without bacteriological confirmation in the NAAT/Xpert era as compared to data provided for 2005-2011 (Publique OFdlS, 2013). Thirdly, because of the restrictive design of our study, sample size is limited. Finally, these results are representative of TDG in a specific setting: the organism burden is dependent on local epidemiology, socio-economic status of the population, health care system, access to care and average time to diagnosis (Auer et al., 2018).

## Conclusion

This study does not corroborate previous observations suggesting that the vast majority of clinical samples cultures for mycobacteria become positive after 4 weeks. In fact, in a high proportion of samples (43%), TDG exceeded 4 weeks. It must however be taken into account that we restricted our analysis clinical samples for which Xpert was either negative or unavailable, for clinical relevance. Case selection thus differs from previous studies. Based on this observation, when a presumptive diagnosis of TB cannot confirmed within hours by NAAT, it is mandatory to wait for at least 8 weeks before reasonably considering cultures as negative. Identification of AFB, cavitation, having an extra-thoracic *vs* pulmonary form of TB, and the combination of these elements all affect significantly TDG.

## Data Availability Statement

The raw data supporting the conclusions of this article will be made available by the authors, without undue reservation.

## Ethics Statement

The study protocol was approved by the ethics committee of the Geneva University Hospitals (CCER 2020-00872) and registered at Clinicaltrials.gov (NCT04718571). Written informed consent for participation was not required for this study in accordance with the national legislation and the institutional requirements.

## Author Contributions

RV-M was implicated in the conception and design of the study, data processing, writing and revision of the manuscript; she approved the final version and agrees to be accountable for all aspects of the work including its accuracy and integrity. AP was implicated in the conception and design of the study, data processing, methodological support and statistics, writing and revision of the manuscript; he approved the final version and agrees to be accountable for all aspects of the work including its accuracy and integrity. GR was implicated in the conception and design of the study, data processing, writing and revision of the manuscript; he approved the final version and agrees to be accountable for all aspects of the work including its accuracy and integrity. JS was implicated in the conception and design of the study, data processing, writing and revision of the manuscript; he approved the final version and agrees to be accountable for all aspects of the work including its accuracy and integrity. J-PJ was implicated in the conception and design of the study, data processing, writing and revision of the manuscript; he approved the final version and agrees to be accountable for all aspects of the work including its accuracy and integrity. All authors contributed to the article and approved the submitted version.

## Funding

This work was supported by the Pulmonary League of Geneva, a non-profit health-care provider involved in patients with chronic pulmonary disorders and mycobacterial infection.

None of the authors received any financial support. The Pulmonary League of Geneva was not implicated in the conception, content or writing of the manuscript.

## Conflict of Interest

The authors declare that the research was conducted in the absence of any commercial or financial relationships that could be construed as a potential conflict of interest.

## Publisher’s Note

All claims expressed in this article are solely those of the authors and do not necessarily represent those of their affiliated organizations, or those of the publisher, the editors and the reviewers. Any product that may be evaluated in this article, or claim that may be made by its manufacturer, is not guaranteed or endorsed by the publisher.
